# Effectiveness of small-angle episiotomy on incisional laceration rate, suturing time, and incisional bleeding in primigravida: A meta-analysis

**DOI:** 10.3389/fmed.2023.1126670

**Published:** 2023-03-07

**Authors:** Yan Zhang, Jiaoyan Zhang, Liang Zhao, Lin Xiao, Jinhui Tian, Wei Fan

**Affiliations:** ^1^Gansu Provincial Central Hospital, Lanzhou, China; ^2^Evidence-Based Medicine Center, School of Basic Medical Sciences, Lanzhou University, Lanzhou, China; ^3^Evidence-Based Nursing Center, School of Nursing, Lanzhou University, Lanzhou, China; ^4^School of Nursing, Southern Medical University, Guangzhou, China; ^5^Key Laboratory of Evidence-based Medicine and Knowledge Translation of Gansu Province, Lanzhou University, Lanzhou, China

**Keywords:** episiotomy, angle, primigravida, meta-analysis, randomized controlled trial

## Abstract

**Objective:**

To investigate the effect of small-angle lateral perineal incision on postoperative perineal rehabilitation in primiparous women.

**Method:**

The Cochrane Library, PubMed, Embase, CINAHL, CNKI, WanFang, VIP, and the Chinese Biomedical Literature Database were searched for randomized controlled trials (RCTs) on the effect of small-angle episiotomy on postoperative maternal perineal wound rehabilitation in puerpera until April 3, 2022. Two researchers independently performed literature screening, data extraction and evaluation of risk of bias in the included literature, and statistical analysis of the data was performed using RevMan 5.4 and Stata 12.0 software.

**Result:**

A total of 25 RCTs were included, with a total sample of 6,366 cases. Meta-analysis results showed that the use of small-angle episiotomy reduced incisional tearing [*OR* = 0.32, 95% *CI* (0.26, 0.39)], shortened incisional suture time [*MD* = −4.58 min, 95% *CI* (−6.02, −3.14)] and reduced incisional bleeding [*MD* = −19.08 mL, 95% *CI* (−19.53, −18.63)], with statistically significant differences (all *p* < 0.05). There was no significant difference in the rate of severe laceration between the two groups [*OR* = 2.32, 95% *CI* (0.70, 7.70), *p* > 0.05].

**Conclusion:**

The use of a small-angle episiotomy during vaginal delivery can reduce the incision tear rate without increasing the incidence of severe perineal laceration, while shortening the incisional suturing time and reducing incisional bleeding. It can be used clinically according to birth canal conditions of the maternal, the intrauterine condition of the fetus and maternal needs.

**Systematic Review Registration:**

PROSPERO International Prospective Register of Systematic Reviews [CRD42022369698]; [https://www.crd.york.ac.uk/PROSPERO/display_record.php?RecordID=369698].

## 1. Introduction

A lateral episiotomy is to cut the perineum at 45° (60° ~ 70° for a highly dilated perineum) from the midline of the posterior perineal coalition to one side, with a length of 4 ~ 5 cm (1). In 1920 De Lee first recommended episiotomy as a way to protect the pelvic floor from lacerations and to protect the fetal head from trauma during vaginal delivery (2, 3). For many years, episiotomy was considered to help prevent more extensive vaginal tears during labor and to heal better than natural lacerations (2, 4). Results from two European centres have shown that episiotomy can significantly reduce the number of genital lacerations, especially in the case of vaginal deliveries in advanced maternal age, higher parity occipitoposterior presentation and fetal macrosomia (5). It has been suggested that for every 6° increase in perineal incision angle from the midline, the risk of third-degree perineal tears is relatively reduced by 50% (6). It has also been suggested that narrower incision angles that are too close to the anal sphincter may increase the risk of obstetric anal sphincter injuries (OASIS) (7). When the incision angle is less than 15° or greater than 60°, the risk of severe perineal tears is nine times higher than that of 15°~60° (8). The laceration condition is closely related to the healing, pain, and infection of the lateral incision wound. Based on clinical experience, some domestic researchers have proposed a small-angle (15°~30°) lateral perineal incision, which reduces both the angle of the lateral incision and the length of the incision to a certain extent, and uses the recovery of the perineal wound after delivery as an important indication to assess the effectiveness of this procedure. In this study, we collected randomized controlled studies on small-angle episiotomy from home and abroad, aiming to evaluate the clinical effects of small-angle episiotomy through an evidence-based approach and provide an evidence-based basis for the selection of the angle of lateral incision during vaginal delivery.

## 2. Materials and methods

### 2.1. Literature search strategy

This review followed the PRISMA (Preferred Reporting Items for Systematic Reviews and Meta-Analysis) guidelines (9). The protocol was registered in the PROSPERO database (CRD42022369698) (10).

PubMed, Embase, The Cochrane Library, CINAHL, China National Knowledge Infrastructure (CNKI), Wanfang, VIP and China Biomedical Literature Database were searched for RCTs on the effect of small-angle episiotomy on maternal prognosis, using the combination of subject headings and free words. English search terms include: episiotomy, perineotomy, angle, mediolateral, lateral, etc. The retrieval time limit was from the establishment of the database to April 2022.

### 2.2. Inclusion and exclusion criteria

#### 2.2.1. Inclusion criteria

(I) Study type: Randomized controlled trials (RCTs). (II) Participants: parturients who underwent lateral episiotomy. (III) Interventions: the intervention group used modified small-angle (15° ~ 30°) episiotomy; The control group received conventional episiotomy (45°, 60° ~ 70° when perineal height distension). (IV) Outcomes: including at least one of the following outcome indicators: perineal laceration during labor, incision suture time, incision bleeding.

#### 2.2.2. Exclusion criteria

(I) Literatures not in Chinese and English; (II) Literatures for which the full text cannot be obtained or repeated publications; (III) Literatures with incomplete data or without reporting the above outcome measures.

### 2.3. Literature screening and data extraction

Two researchers (Zhang JY and Xiao L) independently conducted literature screening and data extraction according to the inclusion and exclusion criteria, and then checked each other. In case of any disagreement, it was resolved through discussion or consulting a third researcher to decide. The data extraction mainly included: (I) basic information of the included studies (e.g., title, first author, publication year, etc.); (II) baseline information of the study subjects (e.g., sample size, age, gestational age, etc.); (III) detailed information of the interventions (e.g., lateral incision angle, incision length, suture method, etc.); (IV) outcome indicators of interest and outcome measurement data; (V) key elements of risk of bias evaluation.

### 2.4. Literature quality evaluation

Two researchers independently evaluated the risk of bias of the included RCTS according to the RCT risk assessment tool (11) recommended by the Cochrane Systematic Reviews Manual 5.1.0, and cross-checked the results.

### 2.5. Statistical analysis

Meta-analysis was performed using RevMan5.4 software. Odds ratio (*OR*) was used as the effect analysis statistic for dichotomous variables. Mean difference (*MD*) was used as effect analysis statistic for continuous variables, and 95% confidence intervals (95%*CI*) was given for estimation of all outcome indicators. The χ^2^ test and *I*^2^ were used to quantitatively determine the magnitude of heterogeneity. If *p* > 0.10 and *I*^2^ < 50%, it indicates that the heterogeneity among the results of each study is acceptable, and meta-analysis was performed using a fixed-effects model (test level *α* = 0.05); If *p* ≤ 0.10 and *I*^2^ ≥ 50% indicated that there was significant statistical heterogeneity among the results of each study, meta-analysis was performed using a random-effects model. Subgroup analysis was selected to find sources of heterogeneity. Egger linear regression in Stata 12.0 software was used to test for publication bias, and the presence of publication bias was suggested if *p* < 0.05.

## 3. Results

### 3.1. Literature screening process and results

**Figure 1:**
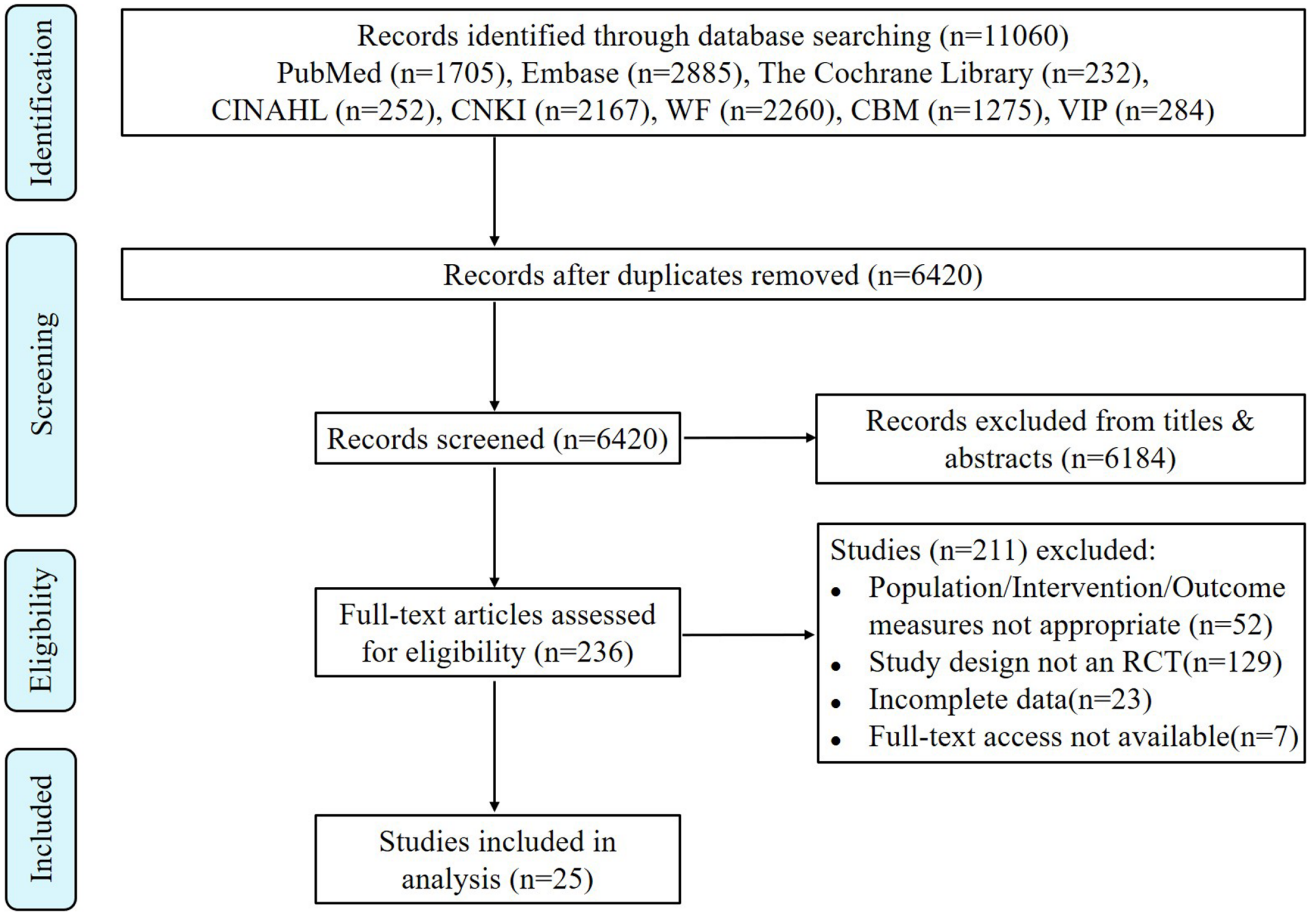
Flow chart of literature screening.

A preliminary search was conducted to obtain 11,060 literature articles. After layer-by-layer screening, 25 RCTs (12–36) were finally included, with a total of 6,366 parturients. The literature screening process and results were shown in Figure 1.

### 3.2. Basic characteristics of included studies

The basic characteristics of the included studies were shown in Table 1. The sample sizes ranged from 50 to 678 cases. The lateral incision angles in the Chinese literature were the comparison between small-angles (15° ~ 30°) and conventional angles (45°), and the lateral incision angles in the English literature were the comparison between 40° and 60° at crowning, i.e., 22.5° versus 45° at non-crowning (37, 38). Eleven studies (13, 15, 19, 25–28, 30, 33–35) had the same incision length in both groups, two studies (20, 29) did not describe the incision length accurately, one (22) stated only the incision length in the test group, and the remaining 13 studies had an incision length approximately 1–3 cm shorter in the test group than in the control group.

**Table 1:** Basic characteristics of the included studies.

Rank	Study	Sample		Intervention		Suturing method	Age/year	Gestational week/W	Fetal weight	Outcome
		T	C	T	C					
1	El-Din A S 2014	165	165	40°, 20 ~ 55 mm	60°，28 ~ 56 mm	The episiotomy wound was repaired by continuous simple suturing using 2/0 slowly absorbable polyglactin 910 stitches. The skin was repaired using the subcuticular technique.	T:20.87 ± 3.01	–	T:2951.85 ± 239.98	①
							C:21.29 ± 3.38			
									C:2985.89 ± 266.87	
2	Chen YH 2009	341	337	15 ~ 30°, 2 ~ 3 cm	45°(60 ~ 70°when the perineum is highly inflated)，4 ~ 5 cm	Vaginal and perineal wounds were closed with interrupted sutures using absorbable threads. The skin was closed using a continuous intradermal suture technique.	23 ~ 35	–	<3,500 g	②
3	Gu XH 2009	300	300	15 ~ 30°, 2 ~ 3 cm	45°(60 ~ 70°when the perineum is highly inflated), 4 ~ 5 cm	Vaginal and perineal wounds were closed with interrupted sutures using absorbable threads. The skin was closed using a continuous intradermal suture technique.	–	–	<3,500 g	②
4	Huang Y 2014	200	200	25 ~ 30°, 3 ~ 4 cm	45°，4 ~ 5 cm	The episiotomy wound was closed layer by layer using absorbable sutures.	20 ~ 32	38 ~ 42	3,250 ± 150 g	①
5	Jin AY 2016	50	50	30°, 1.7 ~ 3 cm	45°，4 ~ 5 cm	The vaginal mucosal and muscular wounds were closed with interrupted absorbable sutures. The skin was closed with embedding sutures.	T:26.660 ± 3.121	–	<4,000 g	①③
							C:26.180 ± 3.102			
6	Li HX 2010	44	60	15 ~ 30°	45°	The vaginal wound was closed with continuous sutures using absorbable sutures. The subcutaneous tissues and muscles were closed with interrupted sutures. The skin was closed with continuous intradermal suturing.	21 ~ 32	–	3,000 ~ 4,000 g	③
7	Li LJ 2011	100	100	20 ~ 30°, 3 cm	45°，4 cm	The vaginal wound was closed with continuous sutures using absorbable sutures. The subcutaneous tissues and muscles were closed with interrupted sutures. The skin was closed with continuous mattress stitching.	–	–	–	①
8	Ling CL 2013	50	50	20 ~ 30°, 2 ~ 3 cm	45°(60°when the perineum is highly inflated)，4 ~ 5 cm	The vaginal mucosa was closed with continuous sutures using absorbable thread. The perineal musculature was closed with interrupted sutures. The skin was closed with continuous intracutaneous sutures.	Average age: 25	–	–	②③
9	Ni XL 2009	120	80	25 ~ 30°, 3 cm	45°, 3 cm	The vaginal mucosa was closed with continuous locking sutures with absorbable thread, the perineal muscle was closed intermittently and the skin was closed with interrupted mattress sutures with silk thread.	Average age: 24	–	≥3,500 g	①
10	Song QX 2007	110	116	25 ~ 30°, 3 cm	45°, 3 cm	–	21 ~ 32	–	≥3,400 g	①
11	Wu SR 2014	150	150	30°	45°	–	T:21 ~ 36	T:34 ~ 43	–	③
								C:34 ~ 42		
							C:22 ~ 37			
12	Xie FY 2016	50	50	30°, 3 ~ 5 cm	45°, 3 ~ 5 cm	The vaginal mucosa, muscle layer and skin were closed with absorbable sutures.	T:26.09 ± 3.45	T:37 ~ 41	–	②③
							C:25.95 ± 3.27	C:37 ~ 40		
13	Yin YM 2015	58	58	25 ~ 30°, 2 ~ 3 cm	45°, 4 ~ 5 cm	The episiotomy wound was sutured with the same suture material and method.	T:27.6 ± 2.9	T:39.3 ± 1.2	–	②③
							C:27.2 ± 2.4	C:39.2 ± 1.5		
14	Zhang C 2012	200	200	20 ~ 30°, 3 cm	45°, 4 cm	The vagina was closed with continuous sutures using absorbable thread. The subcutaneous tissues and muscles were closed with interrupted sutures. The skin was closed with intradermal sutures.	20 ~ 34	–	2,000 ~ 4,000 g	③
15	Zhang XY 2015	149	149	25 ~ 30°, 3 ~ 4 cm	45°, 3 ~ 4 cm	The vaginal mucosa layer was closed with interrupted sutures, the perineal muscle layer was closed with interrupted mattress sutures with No.0 suture, and the skin was closed with continuous intracutaneous sutures.	26.42 ± 5.94	39.78 ± 2.16	2,600 ~ 4,100 g	①
16	Zhang XD 2017	45	45	30°, 3 ~ 5 cm	45°, 3 ~ 5 cm	The episiotomy wound was closed sequentially using absorbable sutures.	T:29.43 ± 1.44	–	–	②③
							C:29.68 ± 1.36			
17	Zhang XM 2015	170	170	30°, 3 ~ 5 cm	45°, 3 ~ 5 cm	The vaginal mucosa, muscle layer and skin were closed in sequence using absorbable sutures.	24 ~ 33	37 ~ 41	3,500 ~ 4,000 g	②③
18	Zhou YX 2013	86	83	30°, 3 ~ 4 cm	45°, 4 ~ 5 cm	The skin was cosmetically closed subcutaneously using absorbable threads.	T:25.33 ± 4.58	T:37.17 ± 2.17	T:3510 ± 750 g	①②③
							C:25.97 ± 3.16	C:38.21 ± 2.52	C:3310 ± 620 g	
19	Cai F 2021	44	44	30°, 3 ~ 5 cm	45°, 3 ~ 5 cm	The episiotomy wound was closed with absorbable surgical sutures。	T:30.25 ± 2.25	T:40.25 ± 0.13	T:3520 ± 430	②③
							C:30.26 ± 2.26	C:40.27 ± 0.15	C:3530 ± 470	
20	Ding YQ 2013	45	45	15 ~ 30°, 3 ~ 4 cm	45°, 3 ~ 4 cm	The episiotomy wound was closed layer by layer using absorbable surgical sutures.	18 ~ 35	37 ~ 41	3,250 ± 450 g	③
21	Jin SF 2005	100	100	25 ~ 30°, 3 ~ 5 cm	45°, 3 ~ 5 cm	–	23 ~ 30	–	≥3,300 g	①
22	Li CX 2009	293	270	30°，3 ~ 4 cm	45°	Intradermal suturing with absorbable thread.	–	–	–	①②
23	Liu GL 2007	32	78	25 ~ 30°, 2 ~ 4 cm	45°, 4 ~ 5 cm	The vagina was closed with continuous sutures using absorbable threads, the subcutaneous tissues and muscles were closed with interrupted sutures and the skin was closed with continuous intradermal sutures.	23 ~ 32	–	3,300 ~ 4,000 g	②③
24	Si WX 2021	25	25	30°, 3 ~ 5 cm	45°, 3 ~ 5 cm	The vaginal mucosa and muscular skin tissue were sutured with absorbable threads.	T:26.9 ± 1.3	–	–	②③
							C:26.7 ± 1.2			
25	Wang JX 2015	257	257	25 ~ 30°, 3 cm	45°, 3 cm	The labial frenulum was sutured in the mattress suturing. The perineal muscle layer was sutured in the intermittent mattress with No. 1 suture.	T:29.91 ± 6.24	T:39.58 ± 2.32	2,600 ~ 4,100 g	①
							C:26.86 ± 5.32	C:39.87 ± 2.19		

Note: T: Test group; C: Control group; −: Unclear; Outcome: ① perineal tears, ② incision suture time, ③ incision bleeding volume.

### 3.3. Risk of bias evaluation results

**Figure 2:**
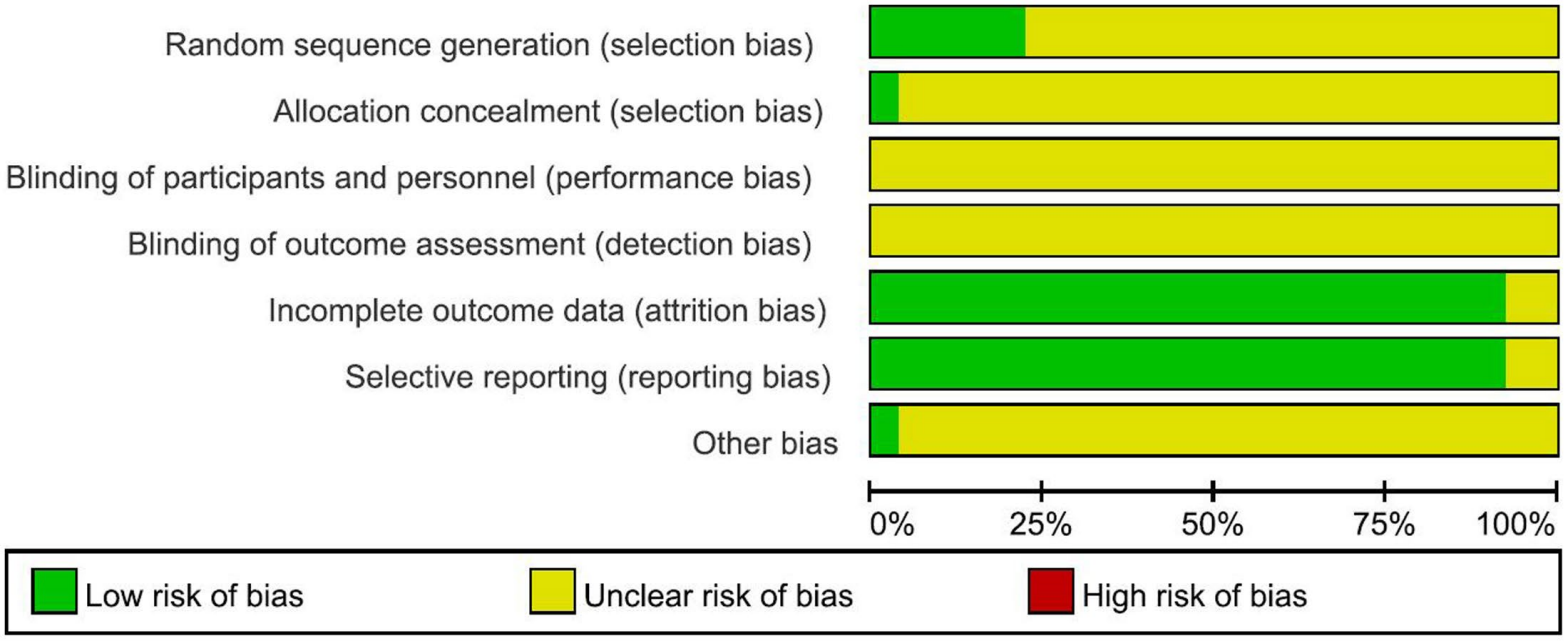
Risk of bias evaluation results.

The evaluation results of the risk of bias of the included studies were shown in Figure 2. According to the risk of bias evaluation criteria recommended by the Cochrane Assist Network, of the 25 included studies, four studies used the randomized number table method (18, 30, 33, 35), two studies (12, 26) used a computerized randomization system for grouping, and the remaining papers only mentioned “randomized” but did not describe the specific randomization method. None of the included studies had missing data, but most of them did not give information about the allocation concealment method and the use of blinded methods.

### 3.4. Meta-analysis results

#### 3.4.1. Incidence of perineal laceration

**Figure 3:**
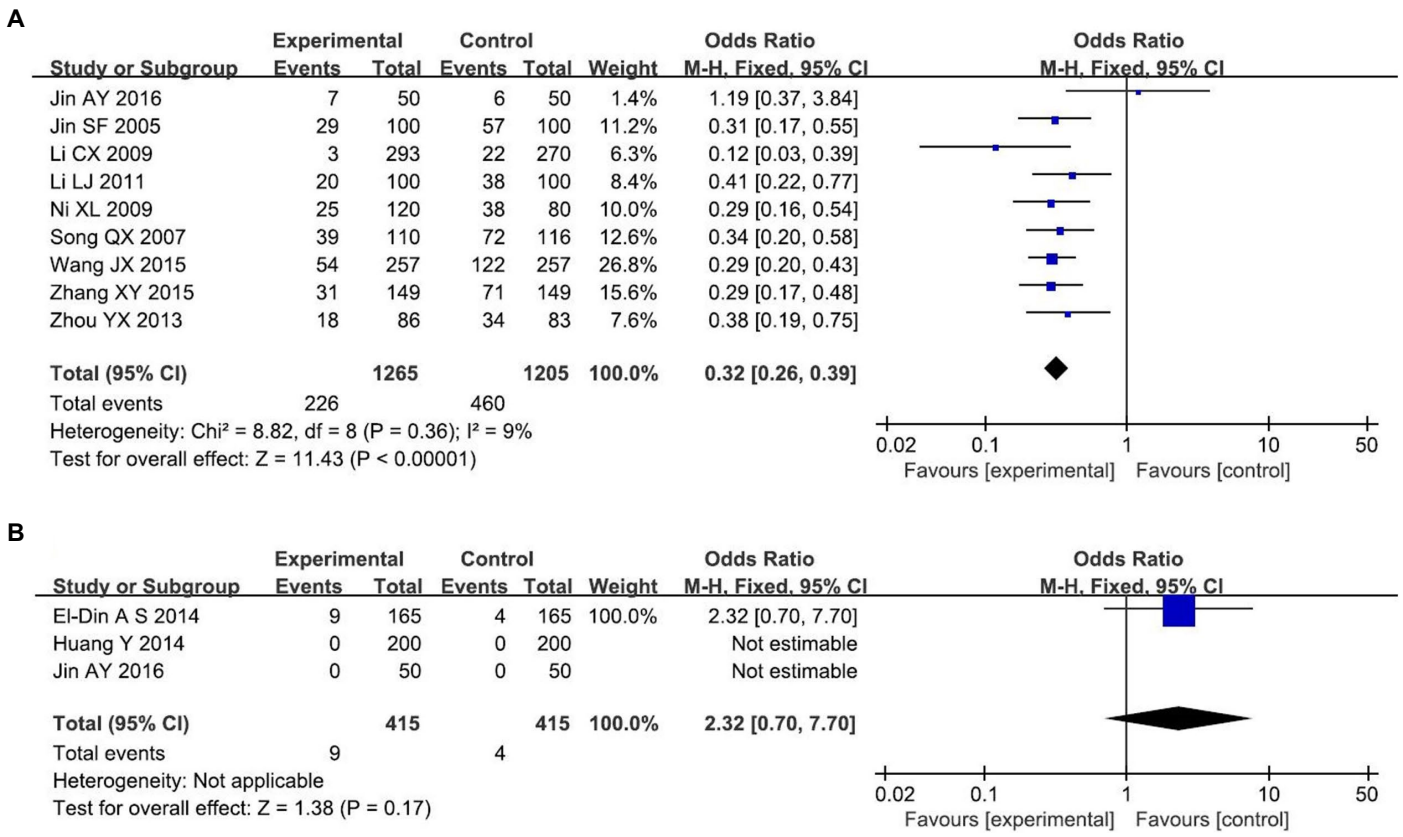
Perineal laceration **(A)** Laceration rate. **(B)** Severe laceration rate.

Nine RCTs (18, 19, 21, 22, 25, 27, 28, 33, 36) reported the effect of lateral incision angle on the rate of lateral incision laceration, including a total of 2,470 primiparas. Fixed-effect model analysis showed a statistically significant difference in the incisional laceration rate in the small-angle perineal lateralization group compared with the conventional perineal lateralization group [*OR* = 0.32, 95% *CI* (0.26, 0.39), *p* < 0.00001], as shown in Figure 3A. Three RCTs (12, 17, 18) reported the effect of lateralization angle on the incidence of severe laceration (perineal third- and fourth-degree laceration) that included a total of 830 parturients. As two of these studies had an incidence of 0 in both groups, a test for heterogeneity could not be performed. EL-Din et al. (12) showed that there was no statistically significant difference between the test and control groups in the rate of severe laceration [*OR* = 2.32, 95% *CI* (0.70, 7.70), *p* > 0.05], as shown in Figure 3B.

#### 3.4.2. Duration of incisional suturing

**Figure 4:**
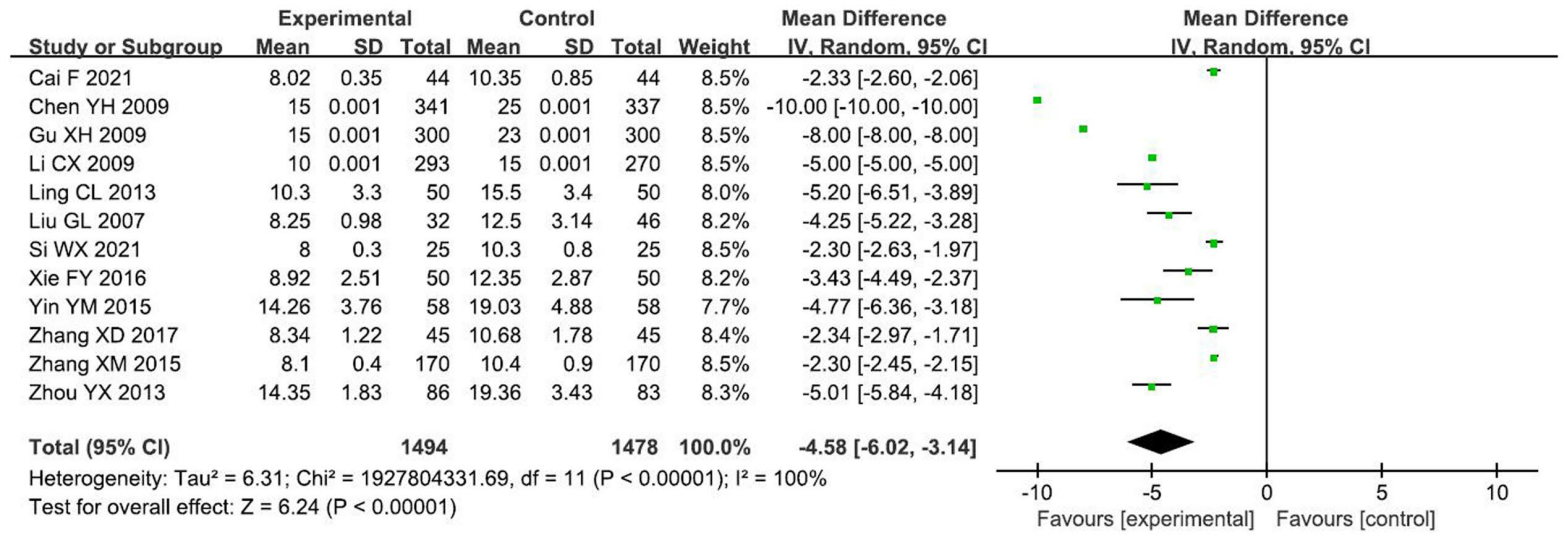
Comparison of incision suture time between the two groups.

A total of 12 RCTs (13, 14, 16, 22–24, 26, 30, 31, 34–36) with 2,972 primiparas were included. Meta-analysis of the random-effects model showed that the incision suture time was significantly lower in the small-angle group than in the conventional group [*MD* = −4.58 min, 95% *CI* (−6.02, −3.14), *p* < 0.00001], with statistically significant differences between the two groups, as shown in Figure 4. Subgroup analysis was conducted according to whether the incision length of the two groups was the same. Five studies had the same incision length in both groups (all 3 ~ 5 cm), with no statistical heterogeneity between studies (*I*^2^ = 8%, *p* = 0.36).so meta-analysis using fixed-effect model showed that the incision suture time in the test group was lower than that in the control group [*MD* = −2.32 min, 95%*CI* (−2.44, −2.20), *p* < 0.00001], and the difference was statistically significant. The incision suture time in other small-angle lateral perineal incisions with different incision lengths was also shorter than that in the control group. As can be seen from Table 2, it is clear that incision length is a source of heterogeneity.

**Table 2:** Results of subgroup analysis comparing the suture time in the two groups.

Outcome indicator	Number of included studies	Heterogeneity test		Effect model	Results of Meta-analysis	
		*I^2^*	*P*		*MD* (95%*CI*)	*P*
Comparison of suture time when the incision lengths were the same in both groups	5	8%	0.36	Fixed	−2.32 (−2.44, −2.20)	<0.00001
Comparison of suture time when the incision lengths were different between the two groups	6	100%	<0.00001	Random	−6.36 (−7.56, −5.17)	<0.00001
Comparison of suture time when the length of the incision in one of the two groups was unclear	1	–	–	–	−5.00 (−5.00, −5.00)	<0.00001

#### 3.4.3. Incisional bleeding volume

**Figure 5:**
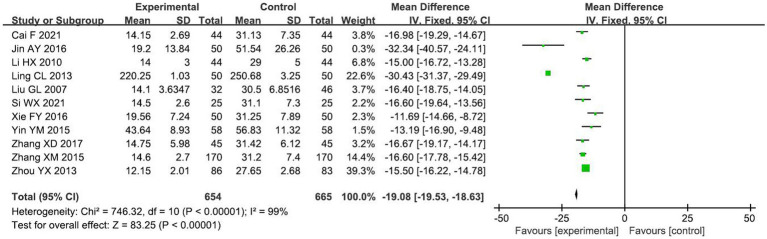
Comparison of incisional bleeding between the two groups.

A total of 11 RCTs (13, 18, 20, 23, 24, 26, 30, 31, 34–36) reported the effect of lateral incision angle on incisional bleeding volume, including 1,319 primigravida. Meta-analysis using a random-effects model showed that incisional bleeding was significantly lower in the small-angle group than in the conventional group [*MD* = −19.08 ml, 95% *CI* (−19.53, −18.63), *p* < 0.00001], with statistically significant differences between the two groups, as shown in Figure 5. The results of the subgroup analysis are shown in Table 3: there were four studies with the same incision length between the two groups, and there was no statistical heterogeneity among the studies (*I*^2^ = 0%, *p* = 0.99), so meta-analysis using fixed-effects model showed that the incisional bleeding volume in the test group was lower than that in the control group [*MD* = −16.67ml, 95% *CI* (−3.18, −2.70), *p* < 0.00001], and the difference was statistically significant; the incisional bleeding volume in other small-angle lateral perineal incisions with different incision lengths were also shorter than those of the control group. From Table 3, it is clear that incision length is a source of heterogeneity.

**Table 3:** Results of subgroup analysis comparing incisional bleeding in the two groups.

Outcome indicator	Number of included studies	Heterogeneity test		Effect model	Results of Meta-analysis	
		*I^2^*	*P*		*OR*/*MD* (95%*CI*)	*P*
Comparison of incisional bleeding when incision lengths was the same in both groups	4	0%	0.99	Fixed	−16.67 (−17.60, −15.75)	<0.00001
Comparison of incisional bleeding when the incision lengths were different between the two groups	7	99%	<0.00001	Random	−19.82 (−20.33, −19.30)	<0.00001

#### 3.4.4. Incisional bleeding >50 mL

**Figure 6:**
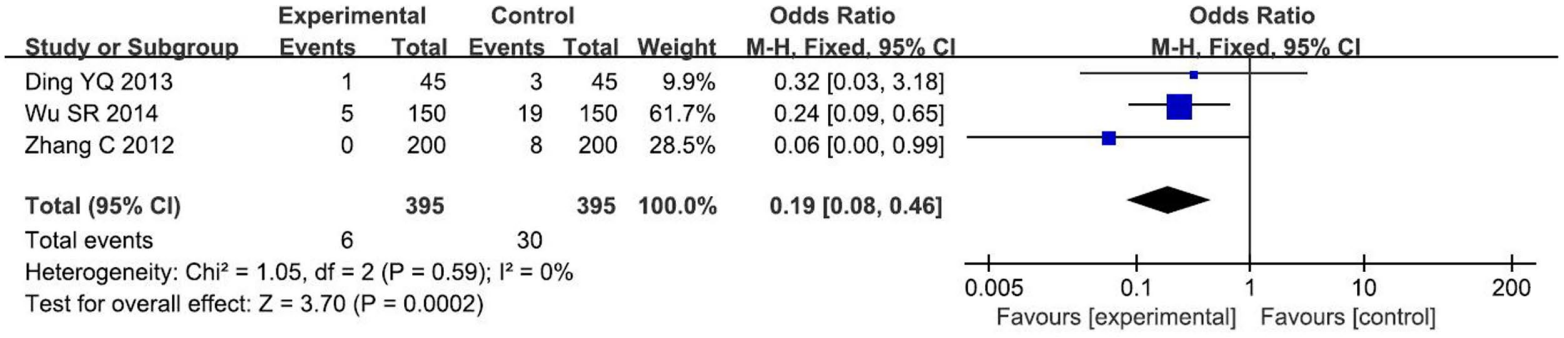
Comparison of incisional bleeding >50 mL between the two groups.

A total of 3 studies (15, 29, 32) with 790 primigravida were included. The incidence of incisional bleeding >50 mL was found to be lower in the test group than in the control group using a fixed-effect model analysis [*OR* = 0.19, 95% *CI* (0.08, 0.46), *p* < 0.00001], and the difference between the two groups was statistically significant, as shown in Figure 6.

### 3.5. Publication bias test

The Egger test in Stata 12.0 software was used to evaluate the publication bias for incisional suture time and incisional bleeding, and the *p* values were greater than 0.05 and the 95% confidence interval included 0, showing that there was no publication bias in the results, as shown in Table 4.

**Table 4:** Egger’s test.

Outcomes	Bias	95%*CI*		*P* > ∣t∣
Suturing time	339.3275	−9971.052	10649.71	0.943
Bleeding volume	2.180309	−9.223097	13.58371	0.676

## 4. Discussion

### 4.1. Small-angle perineal perineotomy reduces the incidence of perineal lacerations and does not increase the incidence of third- or fourth-degree perineal lacerations

The present Meta-analysis showed that small-angle perineal perineotomy did not increase the risk of third- and fourth-degree perineal lacerations, which was basically consistent with the findings of previous studies (39). Episiotomy is intended to prevent severe perineal tears (e.g., OASIS) that may result during transvaginal delivery (40). Some studies have reported that the incidence of OASIS ranges from 0.25 to 7.31% in women who deliver vaginally, and this delivery complication can have a significant impact on maternal health and may lead to a range of problems such as anal incontinence, urinary incontinence, wound infection, perineal pain, sexual dysfunction, and postpartum depression, and a high proportion (42%) of wound complications required further specialist treatment (41–45). This analysis showed a lower rate of tearing with small-angle lateral perineotomy and no substantial difference in the rate of third- or fourth-degree perineal tears. EL-Din et al. (12) found that there was no statistically significant difference in the incidence of third- or fourth-degree lacerations with 40° compared with 60° episiotomy (*p* > 0.05). The reason for this may be that with a larger angle lateral incision, some of the bulbocavernosus and anal raphe muscles will be directly sheared, resulting in a less elastic lateral incision, while the tissue of the inner vaginal wall is more extended and torn due to the larger incision (46). Limited by the small number of included studies, this conclusion needs further confirmation by more studies.

### 4.2. Small-angle episiotomy facilitates rapid closure of the incision in a short time, which can not only shorten the duration of maternal pain and discomfort, but also reduce the degree of postoperative pain

The current study showed that the small-angle lateral episiotomy shortened the suturing time by about 4 min compared with that used in the traditional lateral episiotomy. In the case of greater heterogeneity, the heterogeneity was significantly reduced after removing the studies with different incision lengths in the test and control groups, indicating that the source of heterogeneity may be associated to the length of the incision, which is related to the shortening of the incision length on the one hand and the thickness of the incised tissue on the other hand. The modified lateral episiotomy requires less muscle tissue and vascular tissue to be incised, demonstrating less bleeding, facilitating the recovery of anatomical structures and it is easy to suture, thus significantly shortening the surgical suturing time. In addition, suturing technique is also a factor affecting suture time (47), which certainly includes the suturing skills of the doctor and obstetrician at the time of suturing, and suturing skills can also have a direct impact on episiotomy, while most of the included studies did not specify the suturing method and technique for each layer of tissue, which needs to be further explored in depth in future studies. Future studies need further more comprehensive and in-depth comparisons and studies according to incision length and suturing technique to improve the evidence supporting the effect of episiotomy angle on suture time. The reduction in suture time also correspondingly shortened maternal discomfort during suturing, and the small-angle episiotomy can reduce the incidence of pain in the lateral incision 24 to 72 h postoperatively (39). Postpartum perineal pain has been reported in 92 to 100% of all women, and perineal pain associated with episiotomy or perineal tearing persists in 10% of women, which not only affects the quality of life, but also the persistence of pain may be a cause of postpartum depression (48, 49). Because the small-angle episiotomy only partially cuts the tendon close to the bulbocavernosus muscle, the incision is shallower and shorter, causing less damage to the tissue and correspondingly less postpartum pain. It was also found in this analysis that most of the studies did not specify the assessment method when measuring bleeding, which was not conducive to further comprehensive evaluation by the investigators. It is recommended that future researchers should specify the time of measurement when reporting outcome indicators, so as to provide a reliable basis for evidence-based studies.

### 4.3. A note on the application of small-angle episiotomy

Episiotomy, a widely used invasive procedure in obstetrics, is conditional and complex to perform (50). Major scientific groups, notably the World Health Organization, have explicitly cautioned against routine episiotomy and have reported frequent use of episiotomy without consent because of the additional risks associated with episiotomy, such as infection as well as vaginal discomfort, among others (51, 52). This, along with other controversial and poorly regulated techniques such as hip pressure, has much to do with the definition of obstetric violence (53). These aspects certainly need to be weighed against any benefits associated with episiotomy. Unlike conventional episiotomy, elective episiotomy can avoid the above-mentioned risks to a certain extent (54). Therefore, we are not advocating the routine performance of episiotomy here. In other words, episiotomy should be performed selectively based on clinical judgment and maternal or fetal indications, and must be restricted to those with good reasons (55). On this basis, when elective episiotomy must be performed, we recommend performing it at a small angle to circumvent problems such as perineal laceration and excessive bleeding during vaginal delivery in order to facilitate maternal postoperative perineal recovery. In conclusion, episiotomy remains a common practice, although its use is controversial (56). We need to weigh the risks and benefits of this procedure in a comprehensive manner and use it selectively. It also requires more researchers to further develop high-quality studies in this field to address these controversial issues and promote standardization and science in clinical application.

Limitations of this study: (1) this study is a secondary study, and some of the evidence is limited by the quality of the original studies included; (2) this study only included literature in Chinese and English, and did not involve literature searches in the remaining languages, which limited the extrapolation of the study findings. Future studies could expand the language range to include more high-quality studies to further evaluate the clinical outcomes of small-angle episiotomy.

In conclusion, small-angle episiotomy is beneficial for reducing the incisional tear rate, shortening the incisional suture time, reducing the incidence of incisional infection and incisional pain, and promoting good healing of the perineal incision, which is more beneficial for maternal postoperative recovery. However, due to the limitation of the quality and quantity of included studies, the above findings need to be confirmed by more high-quality studies.

## Data availability statement

The original contributions presented in the study are included in the article/supplementary material, further inquiries can be directed to the corresponding author.

## Author contributions

YZ, JZ, LZ, and JT conceptualized the study, drafted the protocol for the meta-analysis. JZ and LX searched the academic databases and identified the eligible trials. JZ and LZ extracted the data and wrote the initial draft of the manuscript. LZ and LX performed quality assessment. JZ conducted the meta-analysis. YZ, JZ, and LZ interpreted the results. YZ, JZ, LZ, LX, and JT conducted critical review of the manuscript. YZ undertook the post revision and proofreading of the article. FW supervised and reviewed a series of revisions to the article after submission. All authors approved the final version of the manuscript for submission.

## Funding

This study was supported by funding from the Lanzhou Science and Technology Development Guidance Plan Project (2020-ZD-9).

## Conflict of interest

The authors declare that the research was conducted in the absence of any commercial or financial relationships that could be construed as a potential conflict of interest.

## Publisher’s note

All claims expressed in this article are solely those of the authors and do not necessarily represent those of their affiliated organizations, or those of the publisher, the editors and the reviewers. Any product that may be evaluated in this article, or claim that may be made by its manufacturer, is not guaranteed or endorsed by the publisher.
